# Carotid Flow Time Compared with Invasive Monitoring as a Predictor of Volume Responsiveness in ICU patients

**DOI:** 10.24908/pocus.v8i2.16545

**Published:** 2023-11-27

**Authors:** Tomislav Jelic, Jordan Chenkin

**Affiliations:** 1 Department of Emergency Medicine, University of Manitoba Winnipeg, MB Canada; 2 Division of Emergency Medicine, Department of Internal Medicine, Sunnybrook Health Sciences Centre, University of Toronto Toronto, ON Canada

**Keywords:** POCUS, Volume resuscitation, hemodynamic monitoring

## Abstract

**Objectives: **Identifying patients who will have an increase in their cardiac output from volume administration is difficult to identify. We propose the use of carotid flow time, which is a non-invasive means to determine if a patient is volume responsive. **Methods: **Patients admitted to a critical care unit with a pulmonary artery catheter in place were enrolled. We perform a carotid flow time and pulmonary artery catheter measurement of cardiac output pre and post-passive leg raise and comparing the two. An increase of 10% change in the pre- vs. post-passive leg raise measurement would be indicative of a patient who is volume responsive. **Results: **We identified 8 patients who were volume responsive as determined by the gold standard pulmonary artery catheter. The sensitivity 87.5% and specificity 90.9%. Pearson correlation coefficient between PA-CO measurements and CFT was r=0.8316, indicative of strong correlation between the two measurements. **Conclusion: **In our patient sample of critically ill patients with pulmonary artery catheters, we found a strong correlation between corrected carotid flow times and cardiac output measurements from pulmonary artery catheters.

## Background

The use of intravenous fluids to improve cardiac output and restore euvolemia is one of the cornerstones of resuscitation. However, the responsiveness to a fluid challenge is determined by where the patient lies on the Frank-Starling curve. Determining fluid responsiveness can be very challenging when relying on clinical examination or non-invasive measurement 1. Under-resuscitating critically ill patients may cause worsened tissue hypoperfusion and cellular death. On the other hand, the over-resuscitation of these patients can lead to prolonged intensive care stays, ventilator dependence and potentially increased mortality 2, 3.

Carotid flow time (CFT) has been described as a potentially accurate non-invasive method of determining a patient’s fluid responsiveness 4. CFT is a measurement of the duration of time spent in systole and acts as a surrogate for cardiac output. In fluid responsive patients, a fluid challenge will increase the amount of time the heart spends in systole, therefore increasing the CFT. Carotid flow time can be easily assessed using relatively simple measurements at the bedside using portable ultrasound machines. A study by Blehar et al demonstrated that carotid flow time (CFT) changes significantly with the administration of fluids to patients deemed volume deplete 5, further raising the prospect that carotid flow time may indeed be a useful and reliable measurement of volume responsiveness. 

This proof-of-concept study aims to compare the accuracy of CFT for predicting volume responsiveness for patients in the intensive care unit (ICU) when compared with invasive monitoring. 

## Methods

This was a prospective study of patients admitted to an intensive care unit at a large Academic Health Sciences Centre in Toronto, Canada. The institutional review board approved the study. 

A convenience sample of patients were recruited to participate according to investigator availability. Inclusion criteria were ICU patients admitted within the past 24 hours with a pulmonary artery catheter (PAC) inserted for monitoring. This time frame was selected to attempt to capture patients in the most acute phase of their illness. Patients were excluded if they had any cardiac dysrhythmia, known severe valvular lesions and if they were deemed too hemodynamically unstable to participate by intensive care staff. 

Two emergency physicians with significant point of care ultrasound (POCUS) experience enrolled patients in the study. A 10-5 high frequency linear probe (Mindray Medical Ltd, Shenzen, China) was placed over the carotid artery and the carotid bulb was identified. Pulsed wave doppler tracings were obtained with the patient at 30 degrees of head elevation. As previously described, the CFT was measured from the beginning of systole to the dicrotic notch 6. As the CFT measurement was being obtained, a PAC measurement of cardiac output was performed. The ultrasonographer was blinded to the PAC cardiac output measurements. All images were saved for subsequent review. PAC measurements were performed using the thermodilution method. 

After initial measurements were obtained, a passive leg raise was performed as per a standardized protocol 7. A repeat carotid flow time and PAC cardiac output measurement was taken. A total of three CFT and PAC cardiac output measurements were taken during the pre and post-leg raise and were averaged. If there was significant variability (>10%) between the readings of the PAC, five measurements were taken, with the two outlying measurements removed and the three remaining measurements were averaged. The patient’s hemodynamic parameters were also monitored pre and post-passive leg raise. A difference of 10% between the PAC cardiac output readings was considered positive for volume responsiveness as defined in previous literature 8.

A corrected CFT was calculated as systole time/√ cycle time to correct for changes in heart rate. Changes in PAC and CFT cardiac output measurements after the passive leg raise were compared using a two-sided t-test. The changes in CFT were assessed using ROC analysis. Cohen’s kappa coefficient was calculated to assess the agreement between PAC and CFT. Statistical significance was set at p<0.05.

## Results

28 patients were approached for enrollment. Six patients were not enrolled due to consent not being provided by next of kin. One patient was not enrolled as the treating physician felt they were too unstable to participate. A total of 21 patients were subsequently enrolled in the study. Of the 21 patients enrolled, 2 patients were excluded due to a malfunctioning pulmonary artery catheter. Baseline demographics and patient information can be found in Table 1. 

**Table 1:** Patient demographics and baseline data.

**Baseline Patient Demographics**	**Value (%)**
Gender	Male 12 (63.2)
	Female 7 (36.8)
BMI	25.47+/- 1.735
Neck Circumfrence	Mean 46.6cm +/-6.78cm
Reason for PA Catheter *	Shock of unclear etiology 2 (10.5)
	Post CABG 12 (63.2)
	Post Valvular replacement 4 (21.1)
Number of Patients on Ventilator Support	17 (89.5)
Number of Patients on Vasoactive Agents	10 (52.6)
Vasoactive Agents Used	Norepinephrine 3 (14) *two patients receiving both norepinephrine and milrinone
	Milrinone 3 (14) *two patients receiving both norepinephrine and milrinone
	Nitroglycerin 6 (29)
Number of Patients Who Were Volume Responders per Gold Standard	8 (42.1)

The pre and post-leg raise hemodynamic measurements are described in Table 2. Overall, 8/19 (38%) patients exhibited a 10% increase in cardiac output as determined by PAC and were therefore considered volume responsive. 

**Table 2:** Difference Between Pre- and Post- Passive Leg Raise Measurements for Mean Arterial Blood Pressure, Pulse and Corrected Carotid Flow Times for all patients.

**Measurement**	**Pre-Leg Raise**	**Post-Leg Raise**	**p-Value**
Mean Arterial Blood Pressure	84.10 mmHg +/- 20.62	81.95 +/-4.66	0.65
Pulse	87.6 bpm +/- 15.3	87.1 bpm +/- 7.32	0.89
Mean Corrected Carotid Flow Time	303.65ms +/- 32.85	321.85ms +/- 23.56	0.0728
Cardiac Output	4.467 +/- 0.59	5.649 +/- 0.83	0.0678

Table 3 highlights changes in hemodynamics in those who were deemed volume responsive. It is noted that while a change in cardiac output was demonstrated with the CFT and PAC, the patients heart rate and blood pressure did not change significantly, highlighting that an improvement in blood pressure for example, does not necessarily reflect the cardiac output status of the patient.

**Table 3:** Mean difference between pre- and post- passive legraise measurements for mean arterial blood pressure, pulse and correct carotid flow times in volume responders.

-	**Fluid-responsive (n=8)**		**Not fluid-responsive (n=11)**	
**Measurement**	**Pre-leg raise**	**Post-leg raise**	**Pre-leg raise**	**Post-leg raise**
MAP, mmHg (SD)	88.75 (+/-32.06)	82.14 (+/-9.91)	80.45 (+/-8.76)	80.36 (+/-8.96)
Heart rate (SD)	83.75 (+/-13.50)	84.25 (+/-11.02)	89.64 (+/-17.36)	88.27 (+/-18.63)
CFT, ms (SD)	288.85 (+/-42.30)	340.86 (+/-75.86)	310.17 (+/-23.94)	309.57 (+/-29.24)
Change in CFT, ms (SD)	52.01ms (+/-39.96)		14.23ms (+/-10.50)	
CO, L/min (SD)	5.46 (+/-0.93)	6.23 (+/-1.57)	4.52 (+/-1.01)	4.46 (+/-1.07)
Change in CO, % (SD)	0.87 L/min (+/-0.73)		0.21 L/min (+/-0.15)	

The receiver operating curve for carotid flow time measurements had an area under the curve (AUC) of 0.9. The odds ratio is 1.6098, p-value 0.043 (95% CI) (Sensitivity 87.5%, specificity 90.9%, PPV 0.875, NPV 0.909). The ideal change in CFT to predict volume responsiveness was 9.1% change in CFT pre and post passive leg raise. Pearson correlation coefficient between PA-CO measurements and CFT was r=0.8316, indicative of strong correlation between the two measurements. Carotid flow measurements were feasible for all patients, regardless of BMI and neck circumference. 

## Discussion

In this study, CFT was highly accurate at predicting fluid responsiveness among critically ill patients when compared with invasive measurement techniques. Using a change of 9.1%, CFT had a 87.5% sensitivity and 90.9% specificity for predicting fluid responsiveness. Other clinical parameters such as change in heart rate and mean arterial pressure did not change significantly with a fluid challenge. Given its non-invasive nature and easy repeatability, CFT measurements show promise to help guide the clinician on fluid management strategies for critically ill patients. 

The ability of the physician to accurately predict the volume needs of patient remains a difficult clinical skill. With the advent of invasive monitoring, more accurate methods of determining volume status have become available. Over the years however, several of these tools have fallen out of favor, particularly the use of central venous pressure (CVP) and the Swan-Ganz pulmonary artery catheter. Recently, POCUS has been evaluated as a non-invasive means of determining fluid responsiveness. The inferior vena cava (IVC) and its respirophasic variability in the spontaneously breathing patient has shown some promise 9, however there are uncertainties about its utility^10^, ^11^ and ability to accurately predict volume responsiveness. Other methods such as measurements of cardiac output using transthoracic echocardiography can be technically challenging due to the difficulty in obtaining adequate views in critically ill patients 12.

**Figure 1:**
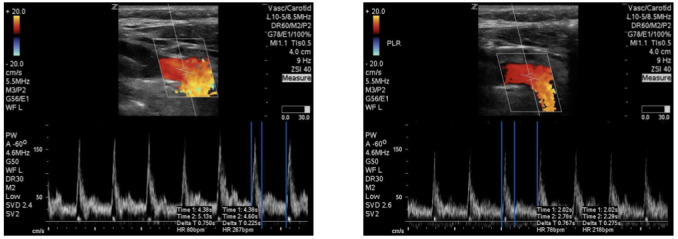
Corrected carotid flow time measurement in a volume non-responder.

**Figure 2:**
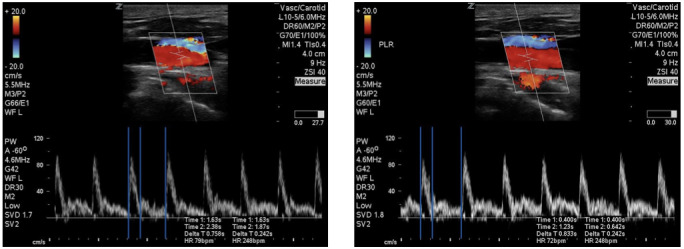
Corrected carotid flow time measurement in volumeresponder .

The rationale for evaluating changes in the CFT is based on common physiologic principles. The CFT measures the time spent in ventricular systole and corrected for changes in heart rate. If a patient is fluid responsive, then their time spent in ventricular systole should increase when faced with a fluid challenge due to increased ventricular filling. This indicates that increased cardiac filling during ventricular diastole results in an increased stroke volume. The time spent is systole is easily measured on a carotid artery pulse waveform from the beginning of the carotid upstroke to the dicrotic notch on the doppler waveform. See Figure 1 for an example of CFT measurement in a volume non-responder and Figure 2 for a volume responder. In the volume responder example, the image on the left is pre-passive leg raise with CFT calculated at 244.05ms. The image on the right is post-passive leg raise with CFT calculated at 314.00ms. This increase of greater than 10% indicated a volume responsive patient that was corroborated by our gold standard.

Recently, the CFT has been evaluated for its potential use in determining fluid responsiveness. In a study by Mackenzie et al in 2015 demonstrated that in blood donors, a demonstrated change in corrected carotid flow time after blood loss with a passive leg raise maneuver 6. A study by Ma et al published in 2017 compared various carotid blood flow measurements to invasive pulmonary artery catheter measurements in non-critically ill patients. Their study found that an average of three waveforms measuring the corrected flow time had a high degree of correlation with cardiac output measurements 13. In 2018, a study by Barjaktarevic et al in a critical care patient population demonstrated that CFT could demonstrate fluid responsiveness compared to a non-invasive gold standard. The study also found that a 7ms change in CFT had a high positive predictive value for identifying patients who will be fluid responder. Barjaktarevic also demonstrated that mechanical ventilation, respiratory rate and variable positive end expiratory pressure had no impact on the performance of CFT 14.

Our study has unique aspects compared to other previously published papers on carotid flow time. First, this study uses a critically ill patient population of which 17/19 were mechanically ventilated and 10/19 on vasoactive agents. Secondly, this study used a invasive means to assess cardiac output with a pulmonary artery catheter. Other published studies used non-invasive means to assess cardiac output 14, which has been shown to be a potentially non-reliable method to assess changes in cardiac output 15.

## Limitations

This study has several important limitations. The sample size was small, which limits the generalizability of our findings. Larger studies should be performed to confirm these findings. Due to investigator availability, a convenience sample was included. However, all eligible patients were approached for enrollment when the investigators were available. Study participants were primarily composed of recent post-operative cardiac surgery patients. These patients may have had unique causes of shock that may not generalize to a broader patient population. We did not measure inter-rater reliability of the CFT measurements. The time taken to complete the scans was not recorded, however it was noted that the measurements could all be completed in less than 10 minutes.

## Conclusion

The corrected carotid flow time has a high accuracy for predicting volume responsiveness in ICU patients when compared with invasive measurements. An increase in CFT of 9.1% was predictive of a significant increase in cardiac output. Future studies with larger and more heterogenous patient populations should be performed to validate these findings. 

## Conflicts of interest

There are no conflicts of interest to disclose for either author. 
